# Multi-drug resistant and extended-spectrum β-lactamases producing bacterial uropathogens among pregnant women in Northwest Ethiopia

**DOI:** 10.1186/s12941-020-00365-z

**Published:** 2020-06-03

**Authors:** Sirak Biset, Feleke Moges, Demeke Endalamaw, Setegn Eshetie

**Affiliations:** Department of Medical Microbiology, College of Medicine and Health Sciences, University of Gondar, P.O. Box: 196 Gondar, Ethiopia

**Keywords:** Extended-spectrum β-lactamase, Multi-drug resistant, Pregnant women, Urinary tract infection

## Abstract

**Introduction:**

Above 80% of urinary tract infections are caused by enteric bacteria, which are known for years by their drug-resistant ability. Though the prevalence of drug-resistant strains is increasing in the world, it is not well known in low-income countries. The aim of this study was to assess the prevalence of Multi-drug resistance, Extended-spectrum β-lactamases production, and associated risk factors among pregnant women in Northwest Ethiopia.

**Methods:**

A hospital-based cross-sectional study was conducted among pregnant women from March to May 2017. A total of 384 clean-catch midstream urine sample was collected from study participants. Bacterial identification and drug susceptibility testing were done following standard microbiological techniques; Extended-spectrum β-lactamase production was screened using a disc diffusion test and confirmed by a combination disc test. The data were entered and analyzed by using SPSS version 20, and a p-value of less than 0.05 was considered as statistically significant.

**Result:**

The overall prevalence of urinary tract infection was 15.9% (95% CI 12.8–20.1%). *E. coli* (49.2%), CoNS (27.9%), and *S. aureus* (18%) were the main uropathogens. The prevalence of MDR uropathogens was 60.65%. The prevalence of ESBLs production among cases caused by *Enterobacteriaceae* was 18.2%. The drug resistance rate of Gram-negative isolates was higher for ampicillin (90.9%), cephalothin (84.8%), and augmentin (57.6%). The drug nitrofurantoin showed the highest activity (100%) against Gram-negative isolates. Gram-positive isolates were showed low susceptibility to penicillin (89.3%) and cotrimoxazole (75%); however highest susceptibility rate for gentamicin (100%), amikacin (100%), and nitrofurantoin (98.36%) was recorded. Prior antibiotic therapy (AOR = 5.46, 95% CI 1.38–21.65) was a risk factor for the presence of multi-drug resistant bacteria.

**Conclusion and recommendation:**

The multi-drug resistance prevalence was high among uropathogen, thus treatment of urinary tract infection during pregnancy; should be based on the antibacterial susceptibility testing result. The isolation of drug-resistant strains like Extended-spectrum β-lactamases in this study calls for the need of periodic and continuous follow-up of antibiotic usage among pregnant women. Nitrofurantoin, gentamicin, amikacin, and ciprofloxacin/norfloxacin showed higher activity against bacterial uropathogen.

## Background check

Urinary tract infection (UTI) is an infection involving urinary tract structures [1]. The urinary tract can be infected in different ways, but the ascent of bacteria from the urethra is the common pathway [2]. Urinary tract infection is the most common public health problem worldwide with women are more likely to be infected due to proximity of the urethral opening to the anus, the presence of vagina, and the shorter length of the urethra [3, 4]. Furthermore, pregnancy facilitates UTIs because of urinary retention in the bladder, increased calyceal and ureteral dilation, and increased levels of urinary glucose and amino acids [5]. Urinary tract infection during pregnancy can cause many complications [6–8]. To prevent these complications, during pregnancy, UTI should be treated [7–12]. However, this high occurrence of UTI in pregnancy could increase the prevalence of inappropriate antibacterial treatment, which in turn increases antibiotic resistance (ABR) [13].

Antibiotic resistance is a growing problem across the world, and indiscriminate use of drugs increases its evolution [14, 15]. It is known that antibiotics are used to clear infection causing bacteria, however, their usage increases the prevalence of antibiotic-resistant strains via selective pressure where antibiotics kill susceptible strains and leave the resistant one to multiply [16, 17]. Production of enzymes is a well-known mechanism that the bacteria are using to resist antibiotics [18, 19]. Extended-spectrum β-lactamases (ESβLs) producing strains can easily hydrolyze β-lactam agents but inhibited by β-lactamase inhibitors [20]. These organisms also carry genes encoding resistance to other classes of antibiotics. As a result, most of them are classified as multi-drug resistant (MDR) organisms [19]. The enzymes and other ABR determinants of a drug-resistant bacteria can also be transferred to drug-susceptible strains [21]. Furthermore, there is a report that these drug-resistant bacteria or genes codes for drug resistance can be transmitted vertically from mothers to their neonates [22]. Urinary tract infections during pregnancy are commonly caused by Gram-negative bacteria, with *Enterobacteriaceae*, in particular, *E. coli* accounted for about 90% of these infections [23]. During pregnancy, untreated UTIs or UTIs caused by MDR or drug-resistant strains like ESβLs are associated with many problems, including the development of pyelonephritis and cystitis in the mother, pre-eclampsia, preterm delivery, low birth weight infant, and cesarean deliveries [24–26]. Multi-drug resistant organisms are associated with history of hospitalization, previous use of any antibiotics, chronic underlying diseases, use of urinary catheters, and previous history of UTIs. [27].

In Ethiopia, UTI among pregnant women is very common with a reported prevalence between 9.2% and 18.8%; *Enterobacteriaceae* are the leading isolates responsible for the infection [28–33]. The WHO global report has revealed the overall epidemiology of ESβL producing bacteria has not been well known in resource-limited countries like Ethiopia [34]. Therefore, this study was aimed at determining MDR and ESβL production in bacterial uropathogens isolated from pregnant women, and identifying associated risk factors for MDR bacterial infection.

## Methods

### Study area, design and period

A hospital-based cross-sectional study was conducted from March to May 2017 at the University of Gondar Hospital (UoGH), Northwest Ethiopia. This referral teaching hospital is one of the biggest hospitals in the Amhara region. About five million people, from the surrounding areas, visit this hospital for different medical services. The study population was pregnant women, who were not on antibiotic treatment for the last 2 weeks from the data collection day, visiting the ANC unit of the hospital.

### Sample size and sampling technique

The sample size of this study was 384. Since there is no clear data on the prevalence of ESβLs producing strains or MDR bacteria among pregnant women, a single population proportion formula, taking p as 50%, was used to calculate the sample size. The systematic sampling technique was applied to select the study participants. Sampling interval was calculated from the sampling frame, estimated number of ANC attendants during the study period. The first study participant was selected using lottery method.

### Data collection procedure

All the study participants were interviewed face to face by trained data collectors (midwives), using a structured and pretested questionnaire, to collect data on the socio-demographic, clinical, and pregnancy-related characteristics. Finally, instruction on how to collect clean-catch midstream urine samples was given to the pregnant women; ~10–20 ml of urine specimen was collected using a sterile screw-capped, wide-mouthed container. The container was labeled with unique code, date, and time of collection then sent to the microbiology laboratory for processing and testing [35].

### Culture and identification technique

The urine specimen was first inoculated on Cysteine-Lactose-Electrolyte-Deficient (CLED) agar using a sterile loop measuring 10 µl then later colonies from CLED agar were subjected to gram staining procedure and then sub-cultured on MacConkey and Blood agar plates (Oxoid, UK). The plates were incubated in the appropriate atmosphere at 37 ℃ for 24–48 h overnight. A colony count of ≥ 10^5^ CFU/ml of urine was considered as significant bacteriuria. The identification of the culture-grown bacteria was based on the gram staining, colony characteristics, and biochemical reactions [35]. The Analytical Profile Index (API 20E) was used to support the bacterial identification process (bioMerieux, France).

### Drug susceptibility testing (DST)

Antibiotic susceptibility testing was carried out using the disc diffusion method. The bacterial suspension was made; its turbidity was adjusted to a 0.5 McFarland turbidity standard before inoculated over the entire surface of Muller Hinton agar [35]. In this study, the following antibiotic discs were used: for Gram-negative bacteria, ampicillin (10 µg), cephalothin (30 µg), nalidixic acid (30 µg), cefepime (30 µg), augmentin (30 µg), ceftazidime (30 µg), cefotaxime (30 µg), cefixime (5 µg), ceftriaxone (30 µg), cefuroxime (30 µg), and cefpodoxime (10 µg); for gram-positive bacteria, cotrimoxazole (1.25/23.75 µg), gentamicin (10 µg), amikacin (30 µg), tetracycline (30 µg), chloramphenicol (30 µg), doxycycline (30 µg), penicillin (10 units), and erythromycin (15 µg), and for both classes of bacteria, cefoxitin (30 µg), norfloxacin (10 µg), ciprofloxacin (5 µg) and nitrofurantoin (300 µg). The zone diameter was interpreted according to the Clinical and Laboratory Standards Institute (CLSI) [36]. Bacterial isolates were regarded as MDR when they were resistant to one or more antibiotics in three or more classes of antimicrobials that the isolate is expected to be susceptible.

### Extended-spectrum β-lactamases detection

All isolates showing reduced susceptibility to either of the 3rd generation cephalosporins (Cefpodoxime zone: ≤ 17 mm; Ceftazidime zone: ≤ 22 mm; Cefotaxime zone: ≤ 27 mm; Ceftriaxone zone: ≤ 25 mm) were selected using disc diffusion method. Those isolates then subjected to a phenotypic confirmatory test for ESβLs detection [37]. A combination disc test was used based on CLSI 2017 recommendations [37]. Cefotaxime (30 µg), ceftazidime (30 µg), ceftazidime-clavulanate (30/10 µg) and cefotaxime-clavulanate (30/10 µg) discs were plated on MHA that was inoculated with a single bacterial suspension. After overnight incubation, 16–18 h at 37 °C, a ≥ 5 mm increase in zone diameter of cefotaxime-clavulanate and/or ceftazidime-clavulanate than the zone diameter of cefotaxime and/or ceftazidime was taken as positive for the presence of ESβLs in bacterial isolates.

### Quality control

A pre-tested and structured questionnaire was used to collect socio-demographic, pregnancy, and clinical-related factors. Standard Operating Procedures (SOPs) were strictly followed during all the stages of sample collection and laboratory work. Reference strains used in this study were *E. coli* ATCC 25922, *P. aeruginosa* ATCC 27853, and *S. aureus* ATCC 25923. The sterility of the prepared media was checked by incubating 5% of the batch overnight at 37 °C. For ESβL positive control, *K. pneumoniae* ATCC 700603, and ESβL negative control, *E. coli* ATCC 25922 strains were used [37].

### Data processing and analysis

The data was entered into SPSS version 20 and was double-checked before analysis. Appropriate descriptive statistics for demographic, clinical, and pregnancy-related characteristics were calculated. The bivariable logistic regression model was fitted for each independent variable. Moreover, those variables having a p-value < 0.2 in univariate analysis were fitted into the multivariable logistic regression model. Odds ratio with a 95% confidence interval and p-values were used to measure the strength of association and to identify statistically significant associated factors, respectively. In multivariable analysis, variables having a p-value < 0.05 were considered to have a statistically significant association with MDR bacterial infection.

## Results

### Socio-demographic characteristics

A total of 384 pregnant women were participated in the present study with the age ranges of 15–45 and a mean (standard deviation) age of 26 (± 5.4). About 349 (90.9%) of the study participants were Orthodox Christians, 305 (79.4%) were urban, and 365 (95%) were married. About 128 (33%) of the participants were illiterate, 167 (43.5%) were housewives, and 110 (28.6%) had monthly income ≤ 1000 birr (Table 1).

**Table 1:** Socio demographic characteristics of the study groups

Socio-demographic characteristics	Frequency	Percentage
Age (in years)		
15–19	21	5.5
20–24	127	33.1
25–29	141	36.7
30–34	51	13.3
> 34	44	11.5
Religion		
Orthodox	349	90.9
Muslim	31	8.1
Protestant	2	0.5
Others	2	0.5
Residence		
Urban	305	79.4
Rural	79	20.6
Marital status		
Currently single	19	4.9
Currently married	365	95.1
Level of education		
Not read and write	128	33.3
Primary	71	18.5
Secondary	99	25.8
College/university	86	22.4
Occupation		
House wife	167	43.5
Farmer	37	9.6
Merchant	46	12
Employed	96	25
Daily labourer	21	5.5
Others	17	4.4
Monthly income (in birr)		
≤ 1000	110	28.6
1000–1800	86	22.4
1801–2800	98	25.5
≥ 2801	90	23.4

### Pregnancy and clinical related factors

Among the study participants, 190 (49.5%) and 119 (31%) were on their 3rd and 2nd trimester, respectively. Based on parity, 224 (58.3%) of the women had more than one pregnancy experience, and 176 (45.8%) of them were nulliparous. Of the total study participants, 26 (6.8%) had a history of prior hospitalization, 62 (16.1%) had a history of previous UTI, and 88 (22.9%) had prior antibiotic therapy. 3.4% of pregnant women had HIV infection. The number of pregnant women with a symptom/s of UTI was 122 (31.8%) (Table 2).

**Table 2:** Pregnancy and clinical related factors among the study group

Pregnancy related characteristics	Frequency	Percentage
Gestation period		
< 3 months	55	14.3
3–6 months	119	31
> 6 months	190	49.5
Don’t know	20	5.2
No of pregnancy		
Prim-gravida	160	41.7
Multi-gravida	224	58.3
No of times giving birth		
Nulliparous	176	45.8
Primiparous	98	25.5
Multiparous	110	28.6
Previous hospitalization	26	6.8
History of UTI	62	16.1
Antibiotic therapy	88	22.9
HIV positive	13	3.4
Participant status for UTI		
Symptomatic	122	31.8
Asymptomatic	262	68.2

### Prevalence of urinary tract infection among the study group

The overall prevalence of UTI among study participants was 61 (15.9% (95% CI 12.8–20.1%)). Of isolated uropathogens, *E. coli* 30 (49.2%) was the commonest uropathogen, followed by CoNS 17 (27.9%) and *S. aureus* 11 (18%). The prevalence of MDR among uropathogens was 37 (60.65%). About 13 (76.47%) of the CoNS and 17 (56.67%) of the *E. coli* isolates were MDR. The prevalence of ESβLs production among *Enterobacteriaceae* was 6 (18.2%). *E. coli* and *Enterobacter cloacae* were the two ESβL producing isolates. Among Gram-positive isolates, 4 (36.36%) of the *S. aureus* were MRSA, and 8 (47.06%) of the CoNS were resistant to methicillin (Table 3).

**Table 3:** The prevalence of MDR, ESβLs producing and MR (Methicillin resistant) bacteria among uropathogens isolated from the study group

Isolates	Number (%)	Frequency and percentage (prevalence)		
		MDR	ESβL	MR
*E. coli*	30 (49.2%)	17 (56.67%)	5 (16.67%)	–
*K. pneumoniae*	2 (3.3%)	1 (50%)	0	–
*E. cloacae*	1 (1.6%)	1 (100%)	1 (100%)	–
*S. aureus*	11 (18%)	5 (45.45%)	–	4 (36.36%)
CoNS	17 (27.9%)	13 (76.47%)	–	8 (47.06%)
Total	61 (100%)	37/61 (60.65%)	6/33 (18.18%)	12/28 (42.85%)

MDR, multi-drug resistant; ESβL, extended spectrum β-lactamase; MR, methicillin resistant

The prevalence of UTI among symptomatic and asymptomatic cases was 38/122 (31.1%) and 23/262 (8.8%), respectively. The Fisher’s exact test showed that history of hospitalization, previous history of UTI, and prior antibiotic therapy were associated with the presence of UTI. The prevalence of MDR bacteria was 25/38 (65.8%) and 12/23 (52.2%) among culture-positive symptomatic and asymptomatic participants, respectively. The prevalence of MDR was significantly higher (p < 0.05) among study participants who had a history of antibiotic therapy than those who hadn’t. (Table 4).

**Table 4:** Pregnancy and clinical related factors against significant bacteriuria and MDR bacteria among pregnant women

Variables	Significant bacteriuria				MDR bacteria			
	Yes	No	Total	p- value	Yes	No	Total	p- value
Gestation period								
< 3 months	6 (10.9%)	49 (89.1%)	55	0.219	3 (50%)	3 (50%)	6	0.808
3–6 months	24 (20.2%)	95 (79.8%)	119		15 (62.5%)	9 (37.5%)	24	
> 6 months	30 (15.8%)	160 (84.2%)	190		18 (60%)	12 (40%)	30	
Don’t know	1 (5%)	19 (95%)	20		1 (100%)	0	1	
No of Pregnancy								
Prim-gravida	29 (18.1%)	131 (81.9%)	160	0.383	21 (72.4%)	8 (27.6%)	29	0.127
Multi-gravida	32 (14.3%)	192 (85.7%)	224		16 (50%)	16 (50%)	32	
No of times giving birth								
Nulliparous	31 (17.6%)	145 (82.4%)	176	0.501	21 (67.7%)	10 (32.3%)	31	0.492
Primiparous	12 (12.2%)	86 (87.8%)	98		6 (50%)	6 (50%)	12	
Multiparous	18 (16.4%)	92 (83.6%)	110		10 (55.6%)	8 (44.4%)	18	
Hospitalization								
Yes	15 (57.7%)	11 (42.3%)	26	*< 0.001*	10 (66.67%)	5 (33.3%)	15	0.807
No	46 (12.8%)	312 (87.2%)	358		27 (58.7%)	19 (41.3%)	46	
History of UTI								
Yes	27 (43.5%)	35 (56.5%)	62	*< 0.001*	20 (74.07%)	7 (25.93%)	27	0.099
No	34 (10.6%)	288 (89.4%)	322		17 (50%)	17 (50%)	34	
Antibiotic therapy								
Yes	27 (30.7%)	61 (69.3%)	88	*< 0.001*	22 (81.5%)	5 (18.5%)	27	*0.007*
No	34 (11.5%)	262 (88.5%)	296		15 (44.12%)	19 (55.88%)	34	
HIV								
Yes	4 (30.8%)	9 (69.2%)	13	0.135	3 (75%)	1 (25%)	4	1
No	57 (15.4%)	314 (84.6%)	371		34 (59.65%)	23 (40.35%)	57	
Participant status for UTI								
Symptomatic	38 (31.1%)	84 (68.9%)	122	*< 0.001*	25 (65.8%)	13 (34.2%)	38	0.433
Asymptomatic	23 (8.8%)	239 (91.2%)	262		12 (52.2%)	11 (47.8%)	23	

The percentage displayed under positive culture and MDR was calculated using the frequency of each classes of the variable group that were enrolled in this study and positive for culture as a denominator, respectively

Italic values means the observed difference is statistically significant (p < 0.05)

MDR, multi-drug resistant; UTI, urinary tract infection; HIV, human immuno-deficiency virus

### Antimicrobial susceptibility pattern

Only 2/61 (3.28%) of bacterial isolates were sensitive to all antibiotics used in this study. The 14 (22.95%), 16 (26.23%), and 19 (31.15%) of the isolates were resistant to two, three, and at least five of the antibiotics tested, respectively (Table 5).

**Table 5:** Distribution of MDR among uropathogens isolated from the study group

Isolated organisms	Number of drugs affected by the isolate						Total
	R0	R1	R2	R3	R4	R5	
*E. coli* (n = 30)	2 (6.67%)	1 (3.33%)	8 (26.67%)	10 (33.33%)	2 (6.67%)	7 (23.33%)	30
*K. pneumoniae* (n = 2)	0 (0.0%)	0 (0.0%)	1 (50%)	1 (50%)	0 (0.0%)	0 (0.0%)	2
*E. cloacae* (n = 1)	0 (0.0%)	0 (0.0%)	0 (0.0%)	0 (0.0%)	0 (0.0%)	1 (100%)	1
*S. aureus* (n = 11)	0 (0.0%)	3 (27.27%)	3 (27.27%)	2 (18.18%)	1 (9.09%)	2 (18.18%)	11
CoNS (n = 17)	0 (0.0%)	2 (11.76%)	2 (11.76%)	3 (17.65%)	1 (5.88%)	9 (52.94%)	17
Total (61)	2 (3.28%)	6 (9.84%)	14 (22.95%)	16 (26.23%)	4 (6.56%)	19 (31.15%)	61

R0, resistant to none (sensitive to all); R1, resistant to one; R2, resistant to two; R3, resistant to three; R4, resistant to four and R5, resistant to five and more antibiotics tested

A high number of *E. coli* isolates were resistant to ampicillin 27 (90%), cephalothin 25 (83.33%), and augmentin 17 (56.7%). None of the Gram-negative isolates were resistant to nitrofurantoin, and none of the Gram-positive isolates were resistant to gentamicin and amikacin. Among the Gram-positive isolates, *Staphylococcus saprophyticus* was the only one resistant to nitrofurantoin. The resistance rate for cefoxitin was 36.4% among *S. aureus*, and 47% among Coagulase Negative *Staphylococcus* (CoNS) isolates (Table 6).

**Table 6:** Distribution of antibiotic resistance among uropathogens isolated from the study group

Antibiotics tested	Isolated organisms (n = 61)					Total (61)	*Enterobacteriaceae* (n = 33)		
	*E. coli* (n = 30)	*K. pneumoniae* (n = 2)	*E. cloacae* (n = 1)	*S. aureus* (n = 11)	CoNS (n = 17)		Non-ESβLs (n = 27)	ESβLs (n = 6)	p-value
AUG	17 (56.67%)	1 (50%)	1	–	–	19/33 (57.6%)	15 (55.6%)	4 (66.7%)	0.618
FEP	6 (20%)	0	1	–	–	7/33 (21.2%)	1 (3.7%)	6 (100%)	*< 0.001*
CFM	6 (20%)	0	1	–	–	7/33 (21.2%)	1 (3.7%)	6 (100%)	*< 0.001*
CRO	5 (16.67%)	0	1	–	–	6/33 (18.2%)	0 (0.0%)	6 (100%)	*< 0.001*
FOX	3 (10%)	0	1	4 (36.36%)	8 (47.05%)	18/61 (26.2%)	1(3.7%)	3 (50%)	0.014
CXM	5 (16.67%)	0	1	–	–	6/33 (18.2%)	0 (0.0%)	6 (100%)	*< 0.001*
AMP	27 (90%)	2 (100%)	1	–	–	30/33 (90.9%)	24 (88.9%)	6 (100%)	0.392
NAL	5 (16.67%)	0	1	–	–	6/33 (18.2%)	2 (7.4%)	4 (66.7%)	*0.005*
CF	25 (83.33%)	2 (100%)	1	–	–	28/33 (84.8%)	22 (81.5%)	6 (100%)	0.556
CPD	5 (16.67%)	0	1	–	–	6/33 (18.2%)	0 (0.0%)	6 (100%)	*< 0.001*
CAZ	5 (16.67%)	0	1	–	–	6/33 (18.2%)	0 (0.0%)	6 (100%)	**< ***0.001*
NOR	4 (13.33%)	0	0	1 (9.1%)	8 (47.05%)	13/61 (21.3%)	2 (7.4%)	2 (33.33%)	0.142
F	0	0	0	0	1 (5.9%)	1/61 (1.64%)	0 (0.0%)	0 (0.0%)	–
CIP	4 (13.33%)	0	0	1 (9.1%)	8 (47.05%)	13/61 (21.3%)	2 (7.4%)	2 (33.33%)	0.142
CTX	5 (16.67%)	0	1	–	–	6/33 (18.2%)	0 (0.0%)	6 (100%)	**< ***0.001*
GEN	–	–	–	0	0	0/28 (0.0%)	–	–	–
PEN	–	–	–	10 (90.9%)	15 (88.2%)	25/28 (89.3%)	–	–	–
TE	–	–	–	2 (18.18%)	9 (52.9%)	11/28 (39.3%)	–	–	–
COT	–	–	–	7 (63.63%)	14 (82.35%)	21/28 (75%)	–	–	–
AMK	–	–	–	0	0	0/28 (0.0%)	–	–	–
DOX	–	–	–	2 (18.18%)	10 (58.82%)	12/28 (42.68%)	–	–	–
C	–	–	–	3 (27.27%)	9 (52.9%)	12/28 (42.68%)	–	–	–
ERY	–	–	–	2 (18.18%)	9 (52.9%)	11/28 (39.3%)	–	–	–

Italic values means the observed difference is statistically significant (p < 0.05)

“–“ Not done; AUG, amox-clavulanic acid; FEP, cefepime; CFM, cefixime; CRO, ceftriaxone; FOX, cefoxitin; CXM, cefuroxime; AMP, ampicillin; NAL, nalidixic acid; CF, cephalothin; CPD, cefpodoxime; CAZ, ceftazidime; NOR, norfloxacin; F, nitrofurantoin; CIP, ciprofloxacin; CTX, cefotaxime; PEN, penicillin; TE, tetracycline; COT, cotrimoxazole; AMK, amikacin; DOX, doxycycline; C, chloramphenicol, and ERY, erythromycin

The ESβLs producing isolates were 100% resistant to all the 3rd generation cephalosporin, cefuroxime, and cefepime. The difference was significant (p < 0.001) when compared to resistance rates by non-ESβLs producing isolates. All the ESβLs and most of the non-ESβLs producing strains were resistant to ampicillin and cephalothin. Uropathogens producing ESβLs were sensitive to nitrofurantoin; most of them were susceptible to fluoroquinolones (Table 6).

The only variable that remained an independent factor for the presence of MDR bacterial infection among pregnant women was a prior history of antibiotic therapy (OR = 5.164, 95% CI 1.226–21.758) (Table 7).

**Table 7:** Bi-variable and multi-variable analysis of factors associated with the presence of MDR bacteria

Variable	MDR		COR (95% CI)	AOR (95% CI)
	Yes	No		
Occupation				
House wife	16	6	5.867 (1.427–24.113)	6.544 (1.005–42.616)
Farmer	7	2	7.700 (1.159–51.171)	4.058 (0.277–59.49)
Merchant	5	4	2.750 (0.509–14.860)	1.781 (0.197–16.066)
Employed	5	11	1	
Daily labourer	2	1	4.400 (0.319–60.614)	1.965 (0.40–97.385)
Others	2	0	–	–
Education				
Not read and write	8	6	2.000 (0.456–8.777)	1.442 (0.169–12.282)
Primary	14	3	7.000 (1.386–35.345)	2.558 (0.287–22.089)
Secondary	9	6	2.250 (0.522–9.697)	0.915 (0.135–6.194)
College and above	6	9	1	
No of pregnancy				
Prim-gravida	21	8	1	
Multi-gravida	16	16	0.381 (0.131–1.110)	0.414 (0.092–1.855)
History of UTI				
Yes	20	7	2.857 (0.959–8.516)	2.687 (0.599–12.052)
No	17	17	1	
Antibiotic therapy				
Yes	22	5	5.573 (1.706–18.205)	5.164 (*1.226–21.758*)*
No	15	19	1	

Italic value means there is a statistical significance between independent and dependent variables (95% CI excludes the null value, which is 1)

COR, crude odd ratio; AOR, adjusted odd ratio; CI, confidence interval

(*) p-value < 0.05

## Discussion

The high prevalence of UTI during pregnancy and fear of its complication on both mothers and their baby [6, 26] encourages the irrational use of antibiotics [38], especially in the developing world where empirical treatment is prevalent. Knowing the prevalence of drug-resistant uropathogens among pregnant women could help the clinicians to improve the wise use of antibiotics during pregnancy.

The prevalence of UTI (15.9%) in this study was in agreement with previous studies in eastern Ethiopia (14%) [33] and in Tanzania (14.6%) [39]. But it was higher than the studies conducted in Bahir Dar (9.5%) by Tazebew D et al. [31] and in Gondar (10.4%) by Alemu et al. [29]. This difference might be due to the higher proportion of symptomatic study participants in the present study.

The most prevalent uropathogen identified was *E. coli* 49.2%, followed by CoNS 27.9% and *S. aureus* 18.03%. Previous reports from different regions of Ethiopia have also reported similar findings [29, 31, 33]. The result, however, was slightly different from the study from Hawassa [28], where CoNS were frequently isolated. This difference might be due to the difference in hygienic practices between study participants and climate and temperature factors.

In the present study, the prevalence of MDR bacteria was 60.65%. This study reported a lower prevalence than previous studies in the same study area, 68% in 2002 [40], and 95% in 2012 [29]. The lower prevalence may be due to differences in MDR definition and/or the antibiotics used for AST. Our prevalence was higher than the study result from Pakistan, where MDR prevalence was 37.9% [41]. The lower prevalence seen in Pakistan may be due to the presence of male participants in the study.

The present study was also aimed to assess the prevalence of ESβLs production among *Enterobacteriaceae.* Hence, the prevalence of ESβLs was 18.2%. This prevalence was lower than previous reports in Ethiopia, which was between 25 and 38.4% [42–44]. Reasonably, difference in study setting, sampling protocol and study population could have an impact on the isolation rate of ESβLs. Likewise, the finding of this study was lower than the study done in India (25.2%) by Selvakumar et al. [45]. On the other hand, it was higher than the study done in Nepal (7.3%) by Thapa et al. [46] and Pakistan (5.8%) by Iqbal et al. [41]. This difference of prevalence might be due to the difference in number of bacterial isolates, and geographical area.

At species level, this study revealed that the prevalence of ESβLs producing *E.coli* was 16.7% and it is incompatible with a report by Olufunke et al. in Nigeria [21]. This prevalence was also lower than the previous study results in Ethiopia. For instance, 36% and 28.2% of ESβL producing *E. coli* was identified in Jimma by 2012 [42] and 2015 [43], respectively.

In the present study, about 90%, 84.8%, and 57.6% of gram-negative uropathogens were resistant to ampicillin, cephalothin, and augmentin, respectively. Previous reports from different parts of Ethiopia also showed a high resistance rate to ampicillin and augmentin [30, 31]. The possible explanation of ampicillin and augmentin resistance might be due to their extensive use in the health facilities as this may boost the selection of uropathogens harboring the β-lactamase enzyme, which can hydrolyze penicillin.

We reported that drugs like nitrofurantoin, gentamicin, and amikacin were effective against uropathogens, followed by norfloxacin and ciprofloxacin. Thapa et al. [46] reported the same antibiotics as the effective drugs to treat bacterial UTIs. The current study also reported that ESβLs producing bacterial uropathogens were resistant to 9 out of 15 antibiotics tested, especially to 3rd generation cephalosporins. This finding was in line with previous studies done by Onwuezobe et al. in Nigeria [47] and by Kumar Yadav et al. in Nepal [48]. This higher antibiotic resistance rate among ESβLs producing isolates may be because of the presence of plasmid carrying genes other than those coding for ESβLs, which are responsible for resistant to other classes of antibiotics like fluoroquinolones.

In the present study, all ESβLs producing isolates were sensitive to the drug nitrofurantoin. The same thing happened in Nepal by Thapa et al. [46] and Sri Lanka by Dissanayake et al. [49]. This similarity in nitrofurantoin sensitivity rate between studies might be because of its narrow range of clinical indications, which results in less usage compared to other UTI drugs.

History of antibiotic usage was the only variable associated with the presence of MDR uropathogens. The previous study in Ethiopia also reported the use of antibiotics as a risk factor for acquiring MDR bacteria [40]. Other studies were also reported antibiotic usage as a risk factor for the presence of ESβLs producing strains, which are usually MDR [50, 51].

### The limitation and strength of the study

The study was done in the hospital setting so that the result may not be representative of other pregnant women attending other health sectors in the same area. Associated factors for MDR bacterial infection were not fully addressed because of the absence of participants with factors like catheterization, and chronic diseases like diabetes mellitus. The number of isolates was small, and this may affect the estimation of the prevalence of ESβL producing strains among pregnant women.

## Conclusion

Antibiotics like nitrofurantoin, ciprofloxacin, and norfloxacin were effective against gram-negative uropathogens, including ESβL producers. Gentamicin, amikacin, and nitrofurantoin were effective against staphylococcus species, which causes UTI. The prevalence of MDR was high among uropathogens, which calls for the use of AST results to treat UTI during pregnancy. The presence of Multi-drug resistant bacterial infection during pregnancy was associated with prior antibiotic therapy. The isolation of ESβLs producing uropathogens and methicillin-resistant staphylococcus species in this study calls for the need of periodic and continuous follow-up of antibiotic usage during pregnancy. Moreover, conducting molecular studies will help to characterize the various drug-resistant strains.

## Data Availability

The datasets used and/or analyzed during the current study are available from the corresponding author on reasonable request.

## Abbreviations

ABR: Antibiotic resistance
ANC: Anti-natal care
AST: Antimicrobial Susceptibility Testing
ATCC: American Type Culture Collection
CLSI: Clinical and Laboratory Standards Institute
CoNS: Coagulase negative *Staphylococcus*
ESβLs: Extended-spectrum β-lactamases
MDR: Multi-drug resistant
MR: Methicillin resistant
MRSA: Methicillin resistant *Staphylococcus aureus*
SOPs: Standard Operating Procedures
SPSS: Statistical Package for Social Sciences
UTIs: Urinary tract infections
